# Unveiling the physics of microcavity lasers

**DOI:** 10.1038/lsa.2017.91

**Published:** 2017-08-25

**Authors:** William E Hayenga, Mercedeh Khajavikhan

**Affiliations:** 1 CREOL, The College of Optics and Photonics, University of Central Florida, Orlando, FL 32816, USA

**Second-order coherence measurements can unambiguously determine whether a light source is generating coherent radiation, that is, whether it is a laser. In a systematic study reported by Kreinberg *et al.*, this measurement is used to additionally reveal intriguing physics associated with ultra-small resonators, such as the identification of sub- or super-radiance emission.**

Recent years have witnessed a growing interest in the development of wavelength- and sub-wavelength-scale lasers. These advancements are, for the most part, motivated by the prospect of higher direct modulation bandwidths, smaller footprints and lower lasing thresholds^1^. The miniature sizes of the cavities give rise to a set of non-classical phenomena governing light−matter interactions that are either negligible or entirely absent in larger resonators. For example, the enhancement of the spontaneous emission rate (Purcell effect) and a large spontaneous emission coupling to the lasing mode (*β*-factor) are routinely observed in micro- and nanoscale lasers. These effects can profoundly influence a laser’s performance and characteristics. In fact, if the *β*-factor approaches unity (see Figure 1), the laser can become ‘thresholdless^2^.’ In such cases, the onset of lasing is difficult to determine because a nonlinear knee (S-shape) is no longer present in the light−light curve plotted on a log−log scale. A systematic study of the second-order coherence properties of emission from small micropillar cavities, reported by Kreinberg *et al.*^3^, sheds light on some of the intriguing physics associated with light−matter interactions in ultra-small resonator systems.

An unequivocal method of determining whether lasing action occurs is through a careful study of the photon emission statistics^4^. This allows one to monitor the second-order coherence function, *g*^2^(*τ*), where below threshold, the photons are bunched and *g*^2^(0)>1, and above threshold, their statistics follow a Poissonian distribution such that *g*^2^(0)→1. However, even this method has limitations because for nanoscale light sources, the linewidth is relatively broad, thus making the coherence time much shorter than the temporal resolution of most single-photon detectors, especially for those operating in the telecom window. Recently, a method of measuring the second-order coherence of broad-linewidth emission was demonstrated in metallic nanoscale lasers^5^. Nevertheless, *g*^2^(*τ*) measurements are laborious, require expensive equipment and ample time. Thus, there is an interest in finding commonly agreed-upon indicators of lasing that could be applied to a variety of high-*β* lasers.

To illustrate the dilemma associated with clearly identifying the onset of lasing in high-*β* light sources, Kreinberg *et al.*^3^ fabricated several quantum-dot-based micropillar devices with various cavity dimensions, resulting in devices with different quality factors and *β* values. For purposes of comparison, light−light, linewidth, and second-order coherence measurements were performed on all devices. As expected, because of the high *β* of the cavities, the light−light plots provided little insight regarding the lasing threshold. Moreover, the linewidth narrowing in a cavity-enhanced LED showed a trend very similar to that of an actual laser. In fact, this device was only shown not to be a laser after its second-order coherence properties were studied. To further elucidate this behavior, the authors fitted the light−light curves, found the intracavity photon numbers (*n*_p_) for the various micropillars, and then plotted the second-order coherence as a function of *n*_p_. Interestingly, they found that for their cavities, *g*^2^(0) approached unity only in the range of *n*_p_ between 10 and 100. This observation suggests that the threshold is more closely related to *n*_p_ than it is to the narrowing of the linewidth. Furthermore, in one of the devices, *g*^2^(0)>2 was observed, an effect attributed to super-radiance.

These results strongly suggest that the designers of high-*β* lasers may soon find that they need to pay more attention to the second-order coherence properties of these devices. These properties are important not only as a tool for decisively determining the devices’ lasing thresholds but also as a means of identifying methodologies for enhancing the performance of such micro- or nanolaser systems. Additionally, such studies can provide insight into cavity quantum electrodynamics and help to identify light sources with sub- or super-radiance emission.

## Figures and Tables

**Figure 1:**
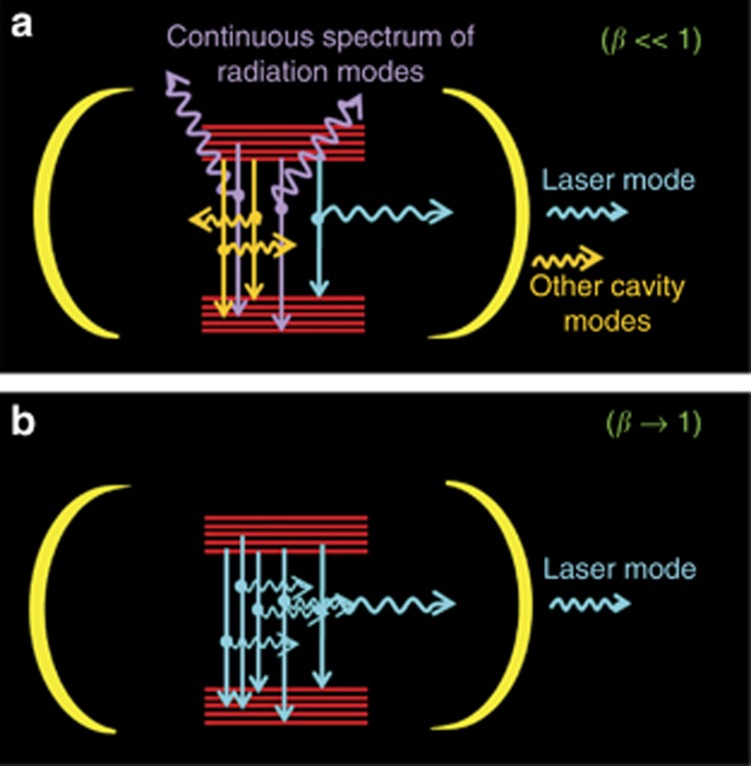
(**a**) A resonator displaying the possible routes the spontaneous emission can follow. It can either be funneled into the lasing mode, be emitted into other cavity modes or go into the continuous spectrum of radiation modes. (**b**) A resonator for a thresholdless laser. If the cavity is judiciously designed, then no other mode lies within the gain bandwidth. This laser is expected to exhibit a spontaneous emission coupling factor of unity.

## References

[bib1] Noda S. Seeking the ultimate nanolaser. Science 2006; 314: 260–261. 17038610 10.1126/science.1131322

[bib2] Yokoyama H. Physics and device applications of optical microcavities. Science 1992; 256: 66–70. 17802593 10.1126/science.256.5053.66

[bib3] Kreinberg S, Chow WW, Wolters J, Schneider C, Gies C et al. Emission from quantum-dot high-β microcavities: transition from spontaneous emission to lasing and the effects of superradiant emitter coupling. Light Sci Appl 2017; 6: e17030. 10.1038/lsa.2017.30 PMC6062317 30167281

[bib4] Mandel L, Wolf E. Coherence properties of optical fields. Rev Mod Phys 1965; 37: 231–287.

[bib5] Hayenga WE, Garcia-Gracia H, Hodaei H, Reimer C, Morandotti R et al. Second-order coherence properties of metallic nanolasers. Optica 2016; 3: 1187–1193.

